# *De novo* design of isopeptide bond-tethered triple-stranded coiled coils with exceptional resistance to unfolding and proteolysis: implication for developing antiviral therapeutics†
†Electronic supplementary information (ESI) available: General materials, methods and the details. See DOI: 10.1039/c5sc02220g


**DOI:** 10.1039/c5sc02220g

**Published:** 2015-08-06

**Authors:** Chao Wang, Wenqing Lai, Fei Yu, Tianhong Zhang, Lu Lu, Xifeng Jiang, Zhenqing Zhang, Xiaoyu Xu, Yu Bai, Shibo Jiang, Keliang Liu

**Affiliations:** a State Key Laboratory of Toxicology and Medical Countermeasures , Beijing Institute of Pharmacology & Toxicology , 27 Tai-Ping Road , Beijing , 100850 , China . Email: keliangliu55@126.com ; Fax: +86-10-68211656 ; Tel: +86-10-6816-9363; b Key Laboratory of Medical Molecular Virology of Ministries of Education and Health , Shanghai Medical College and Shanghai Public Health Clinical Center , Fudan University , Shanghai 200032 , China . Email: shibojiang@fudan.edu.cn ; Fax: +86-21-54237465 ; Tel: +86-21-54237673; c Lindsley F. Kimball Research Institute , New York Blood Center , New York , NY 10065 , USA

## Abstract

Isopeptide bond-tethered triple-stranded coiled coils of HIV-1 gp41 N-terminal heptad repeat (NHR) peptides have been designed with *de novo* auxiliaries to guide site-directed trimerized cross-linking. The presence of isopeptide bridges in the rationally designed trimerization motifs provides extraordinary stability to withstand thermal and chemical denaturation. As a result, these ultra-stable and well-folded trimeric coiled coils direct and yield proteolysis-resistant and remarkably potent N-peptide chimeric trimers with HIV-1 fusion inhibitory activities in the low nanomolar range, much more effective than the corresponding unstructured N-peptide monomers and reaching the potency of clinically used T20 peptide (enfuvirtide). Thus, these isopeptide bond-crosslinked *de novo* coiled coils may also be used as attractive scaffolds for isolating NHR-trimers in other class I enveloped viruses for therapeutic intervention. Furthermore, this isopeptide bridge-tethering strategy could be extendable to the construction of ultra-stable proteins interfering with certain biological processes.

## Abstract 2

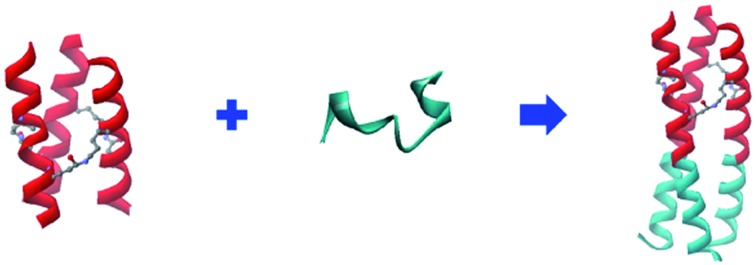
Isopeptide bridge-tethered ultra-stable coiled-coil trimers have been *de novo* designed as structure-directing auxiliaries to guide HIV-1 gp41 NHR-peptide trimerization.

## Introduction

**Fig. 1:**
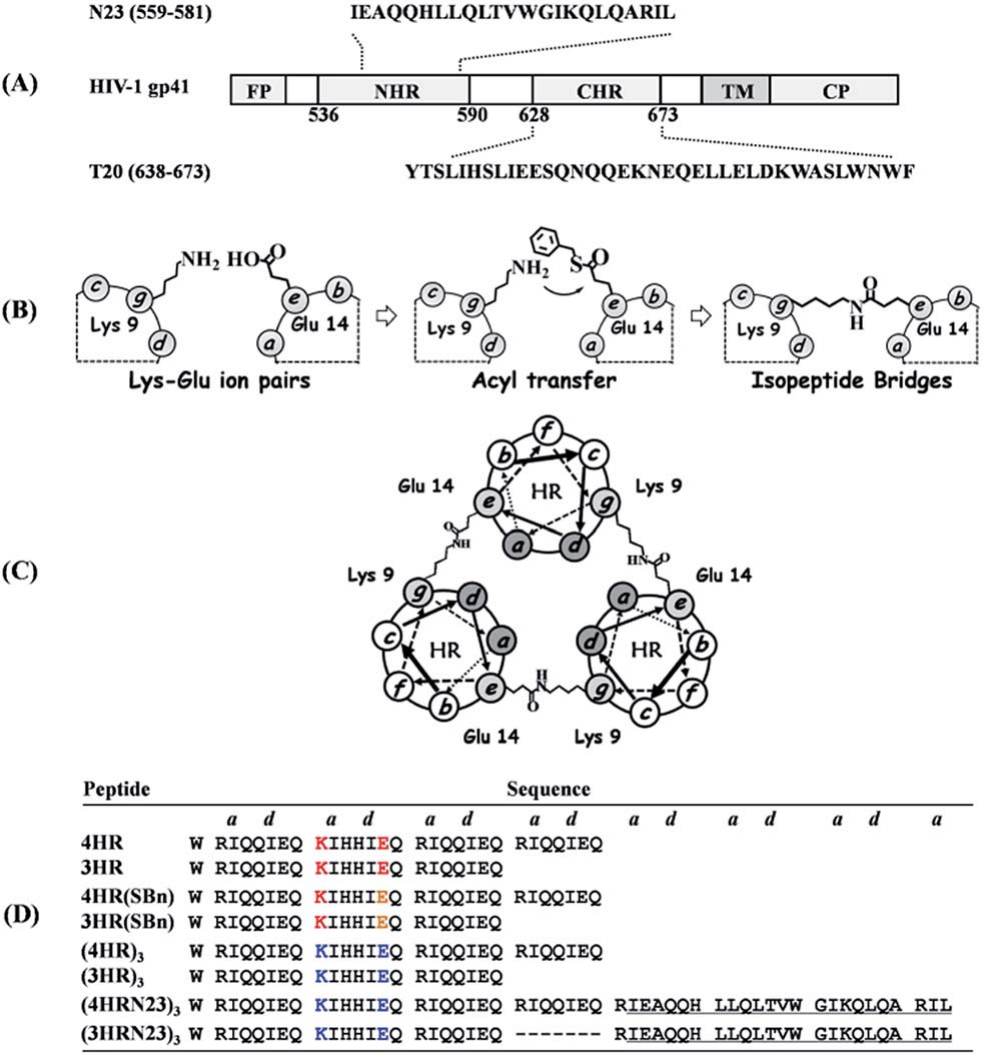
(A) Schematic representation of HIV-1 gp41 and peptidic fusion inhibitors. (B) Schematic representation of isopeptide bond formation *via* an interhelical acyl transfer reaction. For clarity, only one of the three symmetrical active sites is shown. (C) Helical wheel representation of the heptad repeat of cross-linked trimeric coiled coils. (D) Peptide sequences of our designed trimeric coiled coils and chimeric peptides. The specific Lys–Glu interactions are shown in red. The Glu residues with a thioester side chain are highlighted in yellow. Isopeptide bonds are formed between Lys-9 and Glu-14 (in blue). The NHR-derived peptide sequences are underlined.

The coiled coil is a ubiquitous protein folding and assembly motif consisting of two or more amphipathic helices that are wrapped around each other in a superhelical fashion.^1^ Nature provides abundant elegant examples of α-helical coiled-coil proteins with widely ranging functions.^2^ In HIV-1 infection, the formation of a six-helix bundle (6-HB) between a central parallel trimeric coiled coil of N-terminal heptad repeats (NHRs) and the C-terminal heptad repeats (CHRs) of HIV-1 envelope glycoprotein subunit gp41 brings the viral and target cell membranes into close proximity to facilitate their fusion.^3^ The discovery of the 6-HB prompted the search for peptides mimicking the partial structure of CHR and NHR sequences to prevent fusogenic 6-HB formation (Fig. 1A).^4^,^5^ Unfortunately, NHR-derived peptides (N-peptides) in monomeric form show far less potency than CHR-derived peptides (C-peptides), mainly because of their high tendency to aggregate in physiological conditions.^6^ To date, the large number of sequence-to-structure relationship studies in coiled-coil-containing proteins has established several rules of thumb that underline the structure and function of coiled coils.^7^ These basic design principles make coiled coils tractable targets for rational protein design. For example, to avoid the aggregation problem of N-peptides and facilitate their folding into stable and soluble trimers, we previously fused N-peptides with the naturally occurring foldon sequence from T4 fibritin, a highly trimerized motif, to construct potent chimeric N-peptides.^8^ Furthermore, *de novo* designed α-helical coiled-coil peptides that adopt well-defined trimers have also been used in directing and sequestering the gp41 NHR peptide into a nonaggregating trimeric active conformation, thus producing potent N-peptide-based HIV-1 fusion inhibitors.^9^ The introduction of intermolecular disulfide bridges to the N-terminus of trimerization domains has resulted in chimeric N-peptides with dramatically improved thermostability and antiviral activity.^10^ Undoubtedly, disulfide bridges represent an attractive strategy to control and modulate the stability of coiled coil peptides. Generally, disulfide bridges have a potential to be switched between disulfide and thiol due to a change in the redox properties in the biological milieu.^11^–^13^ Furthermore, cystine residues in proteins can undergo destruction *via* β-elimination at elevated temperatures, which also lead to disulfide bridges destabilization.^14^

Isopeptide bonds are known in a variety of contexts, including ubiquitination,^15^ transglutamination,^16^ and Gram-positive pili subunit polymerization.^17^ The presence of isopeptide bonds in bacterial pili suggests that they play a crucial role in maintaining pili integrity in the face of severe mechanical, thermal, and proteolytic stress.^18^ These features make them a new component in Nature's toolbox for stabilizing proteins, thus providing new opportunities for application.^19^ Herein, we focused on generating isopeptide bridge-tethered trimeric coiled coils with exceptional resistance to unfolding *via* an interhelical acyl transfer reaction to investigate their potential for use as trimerization motif in constructing antiviral therapeutics.

## Results and discussion

The triple-stranded coiled coils were designed based on the sequence of Ac-W(R_g_I_a_Q_b_Q_c_I_d_E_e_Q_f_)_*x*_–NH_2_, upon which rationally engineered acyl transfer active sites were incorporated at the helical interfaces to yield site-directed isopeptide bond crosslinks. Ile residues located at every a and d (a–d) position of the heptad favor trimer formation.^20^ In addition, the hydrophobicity of the core also contributes to the overall stability of the assembly.^7^ Favorable g–e′ salt bridge interactions formed between Arg and Glu side chains further stabilized the coiled coils and guided their parallel orientation.^1^ The Glu, Arg, and Gln residues populated the solvent-exposed b–c–f positions to improve solubility. Of note, the specific g–e′ Lys–Glu interhelical ionic interaction at the second heptad was introduced by replacing Arg-9 at the g position with Lys so as to generate juxtaposing acyl donor and acceptor moieties after parallel homo-trimeric assembly formation, and the Glu-14 side chain at the e position was modified with a benzyl thioester (Fig. 1B and S1, ESI†). The Gln residues at both the b and c positions in the second heptad were mutated with His, as His in the active-site microenvironment has been reported to accelerate the acyl transfer.^21^–^23^ Moreover, a Trp was appended to the N-terminus of each sequence to provide a UV chromophore. Following the rationale described above, peptides containing four (*x* = 4) or three (*x* = 3) heptad repeats (HRs) were designed, *i.e.*, 4HR, 4HR(SBn), 3HR, and 3HR(SBn). After acyl transfer occurred specifically, the resulting covalently stabilized trimers, named (4HR)_3_ and (3HR)_3_, respectively, were constructed (Fig. 1C). Thereafter, a portion of gp41 NHR, *i.e.*, N23, was fused to the C-terminus of the trimerized motif without any linking sequence to construct chimeric N-peptide fusion inhibitors (Fig. 1D).

We first used CD spectroscopy to probe the nature and stability of the secondary structure in the *de novo* peptides. Both 4HR and 3HR peptides gave CD spectra consistent with α-helical conformations, characterized by double minima at 208 nm and 222 nm, *i.e.*, with α-helical content of 99.5% and 46.4% folded, respectively. The thermal unfolding transition (*T*_m_) for 4HR and 3HR was determined to be higher than 90 °C and 44 °C, respectively, at 10 μM peptide (Table S1, ESI†). Moreover, their *T*_m_ values were dependent on the peptide concentration, suggesting self-associating species (Fig. S2, ESI†). Therefore, we used sedimentation velocity analysis (SVA) to determine their oligomerization state. Almost all of the 4HR and 3HR peptides at 150 μM showed a sharp peak, with sedimentation coefficients of 1.3 s and 1.2 s, corresponding to 10.5 kDa and 9.9 kDa, which agree with the theoretical molecular masses of the 4HR trimer (11.4 kDa) and the 3HR trimer (8.7 kDa), respectively (Table S1 and Fig. S3, ESI†). Together, these CD and SVA data are consistent with 4HR and 3HR folding to form thermally stable trimeric coiled coils as designed.

Subsequently, we investigated whether 4HR(SBn) and 3HR(SBn) retain the ability to self-assemble into stabilized trimeric coiled coils. CD spectroscopy was applied to compare the α-helicity and thermal stability of the thioester-modified HR peptides with their parent peptides, and it showed that 4HR(SBn) had approximately 17% less helical content than 4HR. Furthermore, 3HR(SBn) only showed 26.3% α-helicity, indicating a poorly folded peptide. Compared with the 4HR- and 3HR-trimers, thermodenaturation analysis of the thioester-modified peptides exhibited a more than 10 °C decrease in the *T*_m_. However, their SVA data were best fit to trimers in solution (Table S1 and Fig. S3, ESI†). Combined, the SVA, CD, and thermal unfolding data indicated that although the thioester mutation weakened the helix–helix interaction, the formation of homotrimeric assemblies brings together acyl-donor and -acceptor moieties to create competent active sites.

**Fig. 2:**
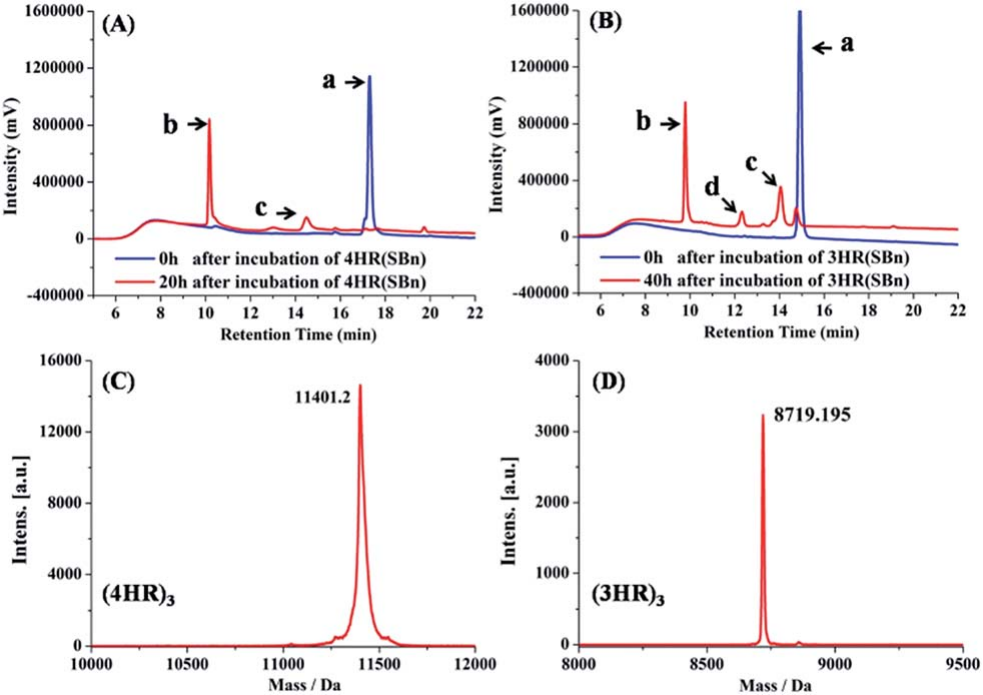
(A) RP-HPLC traces for the acyl transfer reaction of 4HR(SBn) at *t* = 0 and 20 h. a: 4HR(SBn); b: (4HR)_3_; c: incomplete acyl transfer product containing only two isopeptide bonds. (B) RP-HPLC traces for Lys–Glu ligation of 3HR(SBn) at *t* = 0 and 40 h. a: 3HR(SBn); b: (3HR)_3_; c: the hydrolysis product of 3HR(SBn); d: incomplete acyl transfer product containing only two isopeptide bonds. (C) Mass spectrometry of (4HR)_3_ and (D) (3HR)_3_. The MS spectrums in (C) and (D) represent the peaks b in HPLC traces from (A) and (B), respectively.

**Fig. 3:**
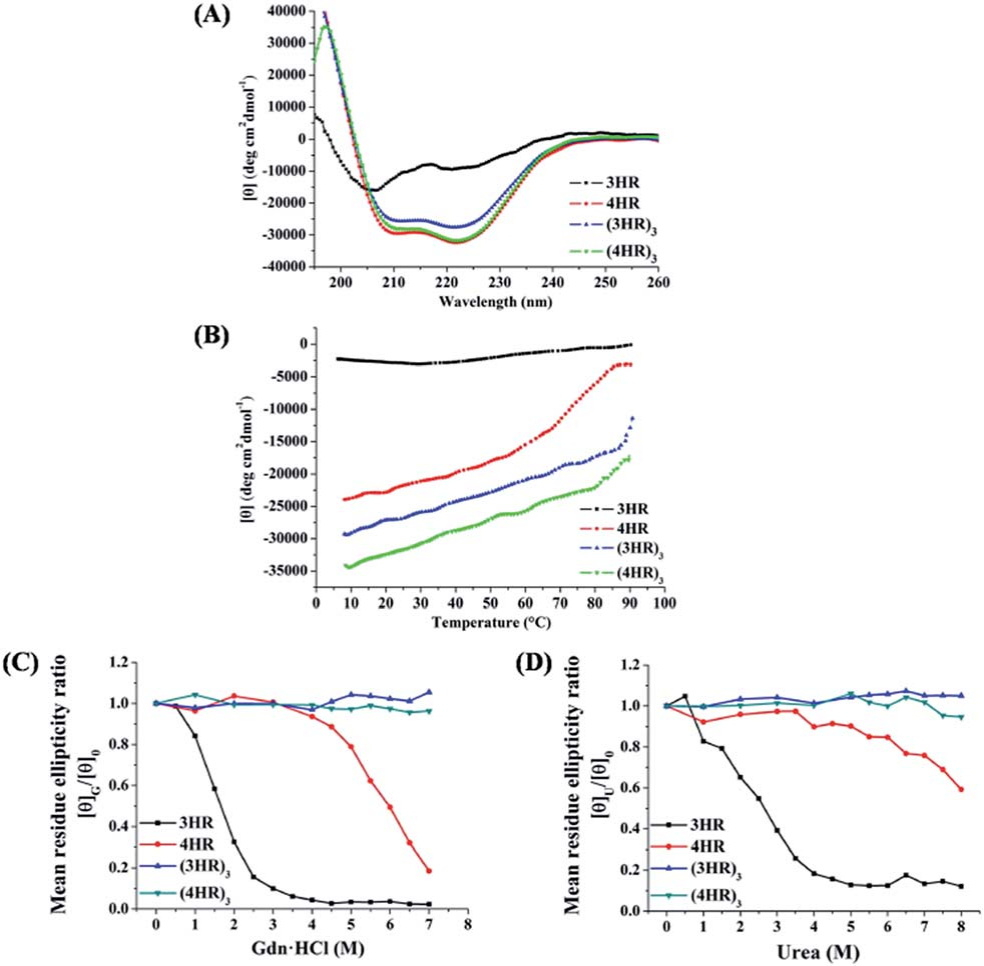
(A) CD spectra of designed trimeric coiled coils in PBS (pH 7.4). (B) Thermal melting profiles of the *de novo* peptides in the presence of 2 M Gdn·HCl. Effect of (C) Gdn·HCl and (D) urea on the ellipticities of the peptides at 222 nm. [*θ*]_G_/[*θ*]_0_: [*θ*]_222_ with Gdn·HCl *vs.* that in the absence of Gdn·HCl; [*θ*]_U_/[*θ*]_0_: [*θ*]_222_ with urea *vs.* that in the absence of urea.

Following the 4HR(SBn)- and 3HR(SBn)-trimer assembly, we studied the isopeptide bond formation by RP-HPLC. HPLC analysis showed a nearly complete reaction of 4HR(SBn) and the appearance of a new peak after 20 h (Fig. 2A). Based on MALDI-TOF-MS analysis, the peak was assigned to the transfer product (4HR)_3_ (Fig. 2C). As shown in Fig. 2B, with a decreased structural stability, the acyl transfer reaction rate of 3HR(SBn) was slower than that of 4HR(SBn), in which the raw material still existed after incubation for 40 h, combined with competing thioester hydrolysis. Furthermore, good agreement between RP-HPLC and Tricine-SDS-PAGE was observed (Fig. S4, ESI†). These results suggested positive correlation between the oligomer stability and acyl transfer rates. Consistent with these data, attempts to generate isopeptide linkages between each pair of engineered acyl-acceptor/donor peptides containing only two copies of HR motifs were unsuccessful because of their inability to adopt the proper tertiary structures (Fig. S5, ESI†). After the acyl transfer reaction completed, the crude mixture was purified by RP-HPLC and the chromatographically isolated (4HR)_3_ and (3HR)_3_ were obtained successfully. The trimer states of (4HR)_3_ and (3HR)_3_ were further confirmed by SVA (Table S1 and Fig. S4, ESI†). The CD spectra of these crosslinked trimeric coiled coils were subsequently analysed. As shown in Fig. 3A, (4HR)_3_ exhibited similar α-helicity with 4HR, while (3HR)_3_ showed 83.4% α-helicity, which was dramatically higher than that of 3HR. Their *T*_m_ values were determined to be higher than 90 °C at 10 μM peptide concentrations (Table S1, ESI†).

In accordance with the general view that isopeptide bonds in cell-surface proteins and protein assemblies add exceptional resistance to unfolding, we would expect to find an increase in coiled-coil stability with the incorporation of isopeptide bridges. The stability of the coiled coils was determined by thermal and chemical denaturation experiments. As shown in Fig. 3B, denaturation of the isopeptide bond-tethered trimers was incomplete, even at 90 °C, in the presence of 2 M guanidinium chloride. These results suggested that the crosslinked trimers were dramatically stable than their corresponding uncrosslinked trimers. In addition, the isopeptide bridge-fixed trimeric coiled coils did not exhibit any concentration dependence to withstand thermal denaturation ranged from 10 °C to 90 °C (Fig. S2, ESI†). Strikingly, chemical unfolding studies painted a similar picture. Fig. 3C shows that (4HR)_3_ and (3HR)_3_ were almost unbreakable in 7 M Gdn·HCl. For 4HR, however, the [*θ*]_G_/[*θ*]_0_ value dramatically decreased to ∼0.2 at 7 M Gdn·HCl, while 3HR completely lost its tertiary structure. In addition, the bridged trimers also exhibited exceptional resistance to 8 M urea denaturation, whereas the [*θ*]_U_/[*θ*]_0_ value of the unbridged trimers abruptly decreased to 0.12–0.59, suggesting their destabilization under chemical stress (Fig. 3D).

These features make (4HR)_3_ and (3HR)_3_ attractive targets as auxiliaries to guide viral NHR region assembly. The chimeric molecules exhibited dramatically improved α-helicity and thermal stability compared with the corresponding free N-peptides (Table S1, ESI†). SVA further confirmed the trimer formation of the chimeric peptides (Table S1 and Fig. S6, ESI†). Subsequently, chimeric N-peptides were tested in parallel with the clinically used T20 peptide for their inhibitory activity against HIV-1 Env-mediated cell–cell fusion and HIV-1 replication. Notably, the corresponding free N23 peptide and trimerized motifs, (4HR)_3_ and (3HR)_3_, had no inhibitory activity at the concentration as high as 10 000 nM, while the chimeric compounds, (4HRN23)_3_ and (3HRN23)_3_, exhibited highly potent anti-HIV-1 activity, reaching the potency of T20 (Table 1). Interestingly, the scaffold length did not correlate with the inhibitory activity, possibly due to the plateau of highly stable tertiary structures. To test the contributions of histidine residues to the peptides' anti-HIV-1 activity, the His residues in the second heptad of (3HRN23)_3_ were substituted with the original Gln residues to construct (3HRMN23)_3_ (Table S2, ESI†). Compared with (3HRN23)_3_, (3HRMN23)_3_ showed similar inhibitory activity in the HIV-1 Env-mediated cell–cell fusion assay, suggesting that the histidine residues in the auxiliaries have no significant influence on the anti-HIV-1 activity of the chimeric N-peptides (Fig. S7, ESI†). However, replacing both His residues with Gln resulted in transfer rate decreases of about 4-fold (Tables S2 and S3, ESI†). In addition, a good agreement between *T*_m_ and EC_50_ was observed, *i.e.*, both crosslinked chimeric N-peptide fusion inhibitors with ultra-high thermostability exhibited 7- to 20-fold more potency than their uncrosslinked counterparts, suggesting that the high structural stability of the isopeptide bridges-tethered constructs contributes positively to their better therapeutic effect (Fig. S8, ESI†).

**Table 1:** Anti-HIV-1 activities of chimeric N-peptides^*a*^

Compound	EC_50_ (nM) for inhibiting		
	cell–cell fusion	HIV–1_IIIB_ infection	HIV-1_BaL_ infection
(4HRN23)_3_	11.6 ± 1.2	51.5 ± 9.3	71.9 ± 7.3
(3HRN23)_3_	10.2 ± 0.9	21.3 ± 1.8	58.2 ± 1.5
(4HR)_3_	>5000	>10 000	>10 000
(3HR)_3_	>5000	>10 000	>10 000
N23	>5000	>10 000	>10 000
T20	14.3 ± 6.6	43.9 ± 9.4	47.9 ± 7.2

^*a*^Peptides were tested in triplicate, and the data are presented as the mean ± standard deviation.

**Fig. 4:**
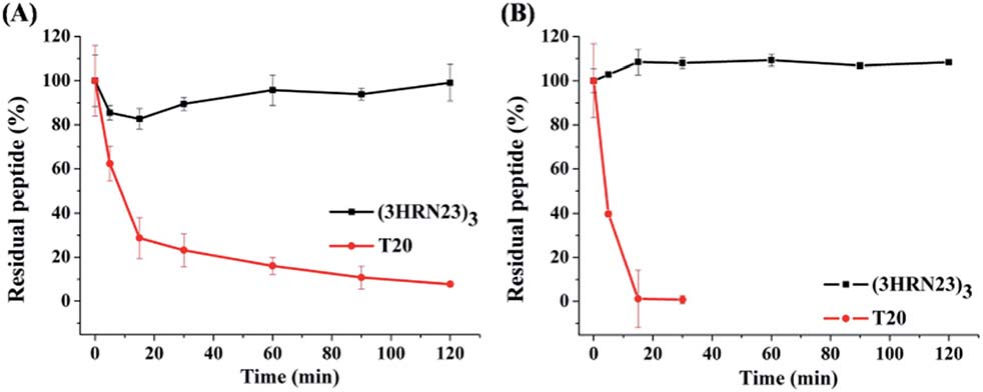
Proteolytic stability of (3HRN23)_3_ and T20 in (A) proteinase K and (B) liver homogenate.

T20 is the first peptidic HIV-1 fusion inhibitor approved for the treatment of HIV-1 infections. Although T20 possesses potent anti-HIV-1 potency, this peptidic drug suffers from short half-life *in vivo* due to its rapid degradation by proteolytic enzymes in the blood, which renders T20 to be s.c. injected twice daily at high dosage (90 mg), resulting painful injection-site reactions.^24^ Strikingly, (3HRN23)_3_ exhibited much more resistant than T20 to proteolytic enzymes (Fig. 4).

## Conclusions

In conclusion, these isopeptide bridge-tethered *de novo* peptides present a well-characterized and stable trimeric coiled coil, even in non-physiological or denaturing conditions. The ultra-high structural stability of the constructs contributes positively to the trimerization of viral NHR segments fused to them as well as the potent and broad HIV-1 inhibitory activity of the chimeras. Since all the class I fusion proteins of the enveloped viruses, *e.g.*, HIV, MERS-CoV, and RSV, share a common feature,^25^ our engineered coiled-coil assemblies may have broader applicability to fuse other NHR peptides, such as those present in other viruses, for the development of antivirals. Furthermore, isopeptide bridges tethering represents an attractive and efficient strategy to control and modulate protein–protein interactions, creating new opportunities for applications in biotechnology.

## Experimental

### Cell–cell fusion assay

Cell–cell fusion assays were performed as described previously.^26^ HL2/3 cells, which stably express HIV Gag, Env, Tat, Rev, and Nef proteins, as well as TZM-bl cells, which stably express large amounts of CD4 and CCR5, were obtained from the NIH AIDS Reference and Reagent Program (contributed by Drs Barbara Felber and George Pavlakis and Drs John C. Kappes and Xiaoyun Wu, respectively). TZM-bl cells (2.5 × 10^4^ per well) and HL2/3 cells (7.5 × 10^4^ per well) were coincubated in 96-well plates (Corning Costar) at 37 °C in 5% CO_2_ in the presence of different concentrations of inhibitors. After incubation for 6–8 h, the medium was aspirated, the cells were washed and lysed, and luciferase activity was measured using the Luciferase Assay System (Promega, Madison, WI) on a SpectraMax M5 plate reader (Molecular Devices, Sunnyvale, CA).

### HIV-1 infection assay

To measure the inhibitory activity of the peptides on infection of HIV-1_IIIB_ and HIV-1_BaL_, 1 × 10^4^ MT-2 cells and TZM-b1 cells, respectively, were infected with 100 times the median tissue culture infective dose of a virus in the presence or absence of the peptides at graded concentrations. On the fourth day post-infection, the culture supernatants were collected for detection of p24 antigen using an enzyme-linked immunosorbent assay (ELISA). The percent inhibition by the peptides and EC_50_ values was calculated using Calcusyn software.^27^

### Circular dichroism (CD) spectroscopy

Lyophilized peptides were resuspended in ddH_2_O (pH 7.0) at a concentration of approximately 1 mg mL^–1^. All the HR-peptides and chimeric N-peptides were diluted in PBS (pH 7.4) to the specific final concentration indicated in the Results and discussion section. The peptides were incubated in a water bath set at 37 °C for 0.5 h before testing. CD spectra were acquired on an MOS-450 system (BioLogic, Claix, France) using the following parameters: band width, 4.0 nm; resolution, 0.1 nm; path length, 0.1 cm; response time, 4.0 s; and scanning speed, 50 nm min^–1^. For CD thermal denaturation analysis, the temperature was controlled by a BioLogic TCU250 system, and CD spectra were monitored at 222 nm from 10 °C to 90 °C at a scan speed of 2 °C min^–1^.

### Sedimentation velocity analysis (SVA)

All measurements were performed on a ProteomelabTMXL-A/XL-I analytical ultracentrifuge (Beckman Coulter, Fullerton, CA, USA) at 20 °C, as described previously. In brief, three-channel cells were used with an An-60 Tirotor. Lyophilized peptides were resuspended in ddH_2_O (pH 7.0) at approximately 1 mg mL^–1^. The individual proteins were prepared in PBS and incubated at 37 °C for 30 min. All samples were prepared at a final concentration of 150 μM and were initially scanned at 3000 rpm for 10 min to identify the appropriate wavelength for data collection. Data were collected at 60 000 rpm at a wavelength of 280 nm. Sedimentation coefficient distribution, *c*(*s*), and molecular mass distribution, *c*(*M*), were calculated using the SEDFIT program.

### Denaturation studies

The stock solutions of the peptides prepared in PBS (pH 7.4) were diluted with calculated volumes of 7.5 M guanidine hydrochloride or 8 M urea dissolved in the same buffer to give the desired final denaturant concentrations. The samples were incubated at room temperature for 3 h before their full-range CD spectra or the ellipticities at 222 nm were recorded. For thermal denaturation studies, the stock peptide solutions were diluted with appropriate volumes of buffer and guanidine hydrochloride. Thermal denaturation was performed by monitoring the ellipticity change at 222 nm from 10 °C to 90 °C at a rate of 2 °C min^–1^.

## Supplementary Material

Supplementary information
