# Supplementary material for: De novo design of isopeptide bond-tethered triple-stranded coiled coils with exceptional resistance to unfolding and proteolysis: implication for developing antiviral therapeutics
Source: Chem Sci. 2015 Aug 6;6(11):6505–9. doi: 10.1039/c5sc02220g (PMC6054081; doi:10.1039/c5sc02220g)
Supplement: Supplementary file 1 [file SC-006-C5SC02220G-s001.pdf]

Electronic Supplementary Material (ESI) for Chemical Science.
This journal is © The Royal Society of Chemistry 2015

Supporting Information

De novo Design of Isopeptide Bond-Tethered Triple-Stranded Coiled Coils with Exceptional Resistance to Unfolding and Proteolysis: Implication for Developing Antiviral Therapeutics

Chao Wang,[‡a] Wenqing Lai,[‡a] Fei Yu,[b] Tianhong Zhang,[a] Lu Lu,[b] Xifeng Jiang,[a] Zhenqing Zhang,[a] Xiaoyu Xu,[a] Yu Bai,[a] Shibo Jiang*[bc] and Keliang Liu*[a]

[a] State Key Laboratory of Toxicology and Medical Countermeasures, Beijing Institute of Pharmacology & Toxicology, 27 Tai-Ping Road, Beijing, 100850, China;
[b] Key Laboratory of Medical Molecular Virology of Ministries of Education and Health, Shanghai Medical College and Institute of Medical Microbiology, Fudan University, Shanghai 200032, China;
[c] Lindsley F. Kimball Research Institute, New York Blood Center, New York, NY 10065, USA;
[‡] These authors contributed equally to this work.

Corresponding Authors:

K.L.: State Key Laboratory of Toxicology and Medical Countermeasures, Beijing Institute of Pharmacology & Toxicology, 27 Tai-Ping Road, Beijing, 100850, China; Tel.: 86-10-6816-9363; Fax: 86-10-6821-1656, E-mail: keliangliu55@126.com.

S.J.: Key Laboratory of Medical Molecular Virology of Ministries of Education and Health, Shanghai Medical College and Institute of Medical Microbiology, Fudan University, Shanghai 200032, China; Tel.: 86-21-54237673; Fax: 86-21-54237465; E-mail: shibojiang@fudan.edu.cn.

Table S1. Biophysical properties of the designed peptides[a]

Compd	Helicity (%)	*Tm* (°C)	Sedimentation coefficient (s)	Observed molecular mass (kDa)	Calculated molecular mass (kDa)
4HR	99.5	> 90	1.3	10.5	3.8[b]
3HR	46.4	44	1.2	9.9	2.9[b]
4HR(SBn)	82.9	86	1.4	11.3	3.9[b]
3HR(SBn)	26.3	32	1.1	10.3	3.0[b]
(4HR)$_3$	96.2	> 90	1.5	13.3	11.4
(3HR)$_3$	83.4	> 90	1.2	9.2	8.7
(4HRN23)$_3$	98.8	> 90	1.9	22.6	19.9
(3HRN23)$_3$	89.6	> 90	1.6	17.3	17.2
N23	16.8	not detected	~	~	~

[a] CD spectra of each designed peptide were monitored in PBS, pH 7.4. The final peptide concentration was 10 μM. Sedimentation equilibrium studies were performed at a concentration of 150 μM in PBS (pH 7.4) and a rotor speed of 60,000 rpm at 20 ºC. [b] The theoretical molecular mass for the HR peptides in the monomeric conformation.

Table S2. Sequences of designed peptides[a]

Compound	Sequence[a]
3HR(SBn)N23	W RIQQIEQ KI**HH**I<u>E</u>Q RIQQIEQ RIEAQQH LLQLTVW GIKQLQA RIL
3HRM(SBn)N23	W RIQQIEQ KI**QQ**I<u>E</u>Q RIQQIEQ RIEAQQH LLQLTVW GIKQLQA RIL
(3HRN23)$_3$	W RIQQIEQ *K*I**HH**I*E*Q RIQQIEQ RIEAQQH LLQLTVW GIKQLQA RIL
(3HRMN23)$_3$	W RIQQIEQ *K*I**QQ**I*E*Q RIQQIEQ RIEAQQH LLQLTVW GIKQLQA RIL

[a] The Glu residues with a thioester side chain are underlined. Isopeptide bonds are formed between Lys-9 and Glu-14 (in italics).

Table S3. The rate constant and $t_{1/2}$ of the inter-helical acyl transfer reaction

Compound	k (10^{-3} s^{-1})	$t_{1/2}$ (h)
3HR(SBn)N23	2.99	4.53
3HRM(SBn)N23	0.76	20.1

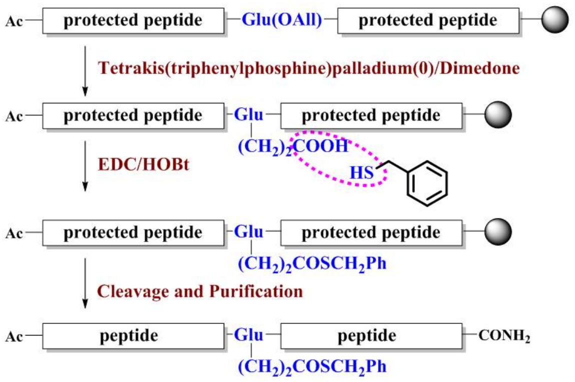

Fig. S1. Strategy for the preparation of thioester-modified peptides.

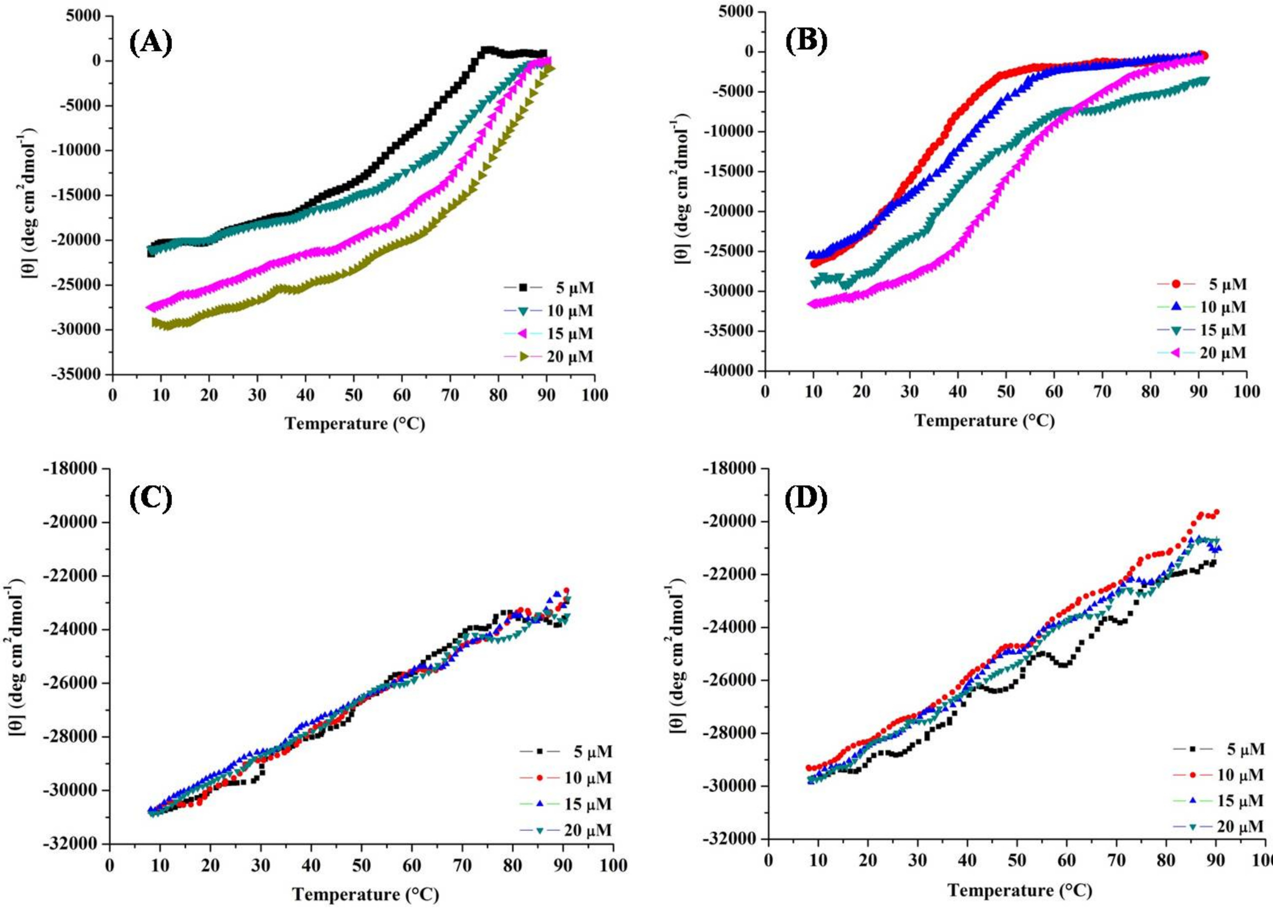

Fig. S2. (**A**) The thermostability of 4HR at different concentrations in PBS (pH 7.4) containing 2M guanidinium hydrochloride and (**B**) 3HR at various concentrations in PBS (pH 7.4). (**C**) The thermostability of $(4HR)_3$ and (**D**) $(3HR)_3$ at different concentrations in PBS containing 4M guanidinium hydrochloride.

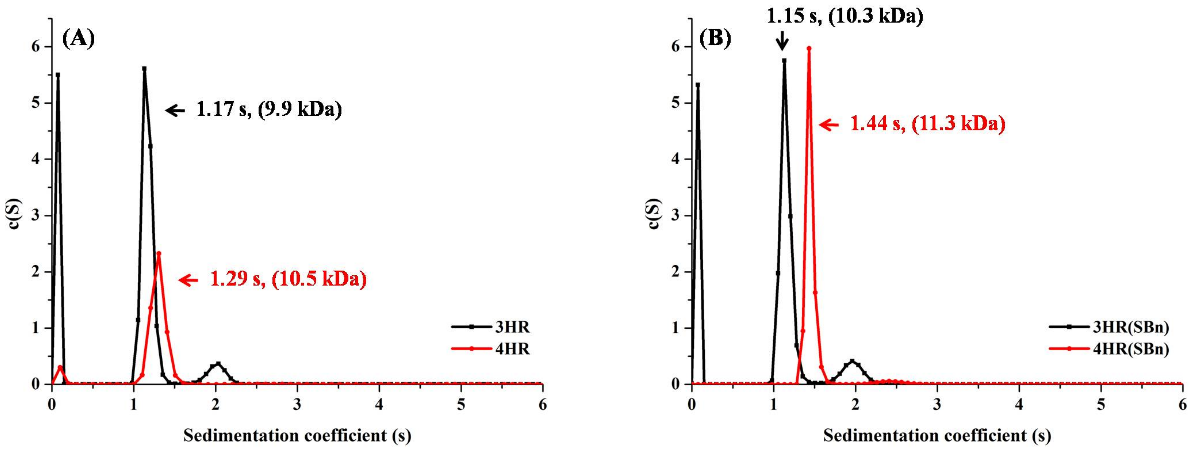

Fig. S3. Sedimentation velocity analysis of (**A**) 4HR and 3HR peptides and (**B**) 4HR(SBn) and 3HR(SBn).

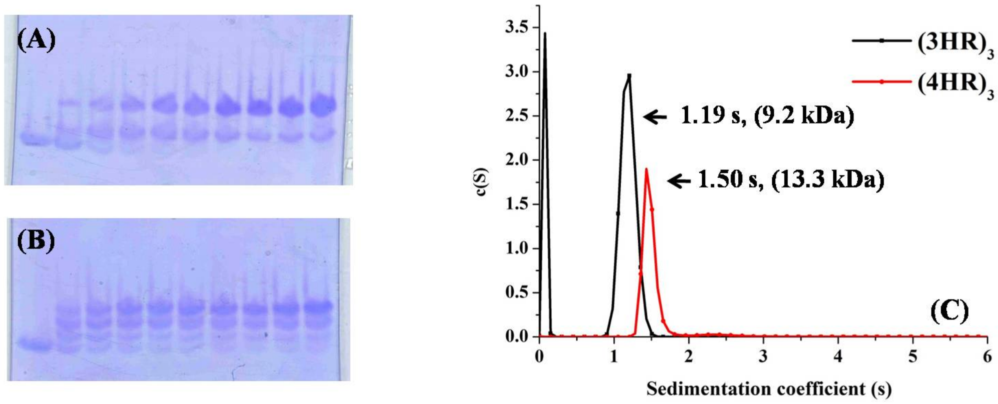

Fig. S4. (**A**) SDS-PAGE analysis of 4HR(SBn) at 0 h (lane 1), 2 h (lane 2), 4 h (lane 3), 6 h (lane 4), 8 h (lane 5), 12 h (lane 6), 14 h (lane 7), 16 h (lane 8), 18 h (lane 9), and 20 h (lane 10). (**B**) SDS-PAGE analysis of 3HR(SBn) at 0 h (lane 1), 4 h (lane 2), 8 h (lane 3), 12 h (lane 4), 16 h (lane 5), 20 h (lane 6), 24 h (lane 7), 28 h (lane 8), 36 h (lane 9) and 40 h (lane 10). (**C**) SVA of crosslinked $(4HR)_3$ and $(3HR)_3$ trimers.

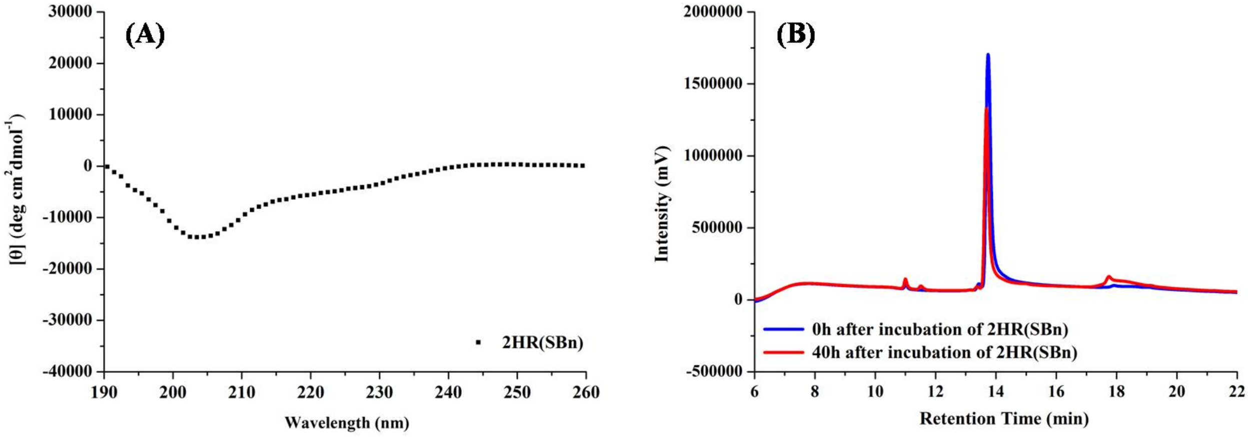

Fig. S5. (**A**) CD spectra of 2HR(SBn). (**B**) RP-HPLC traces of 2HR(SBn) at t=0 and 40 h.

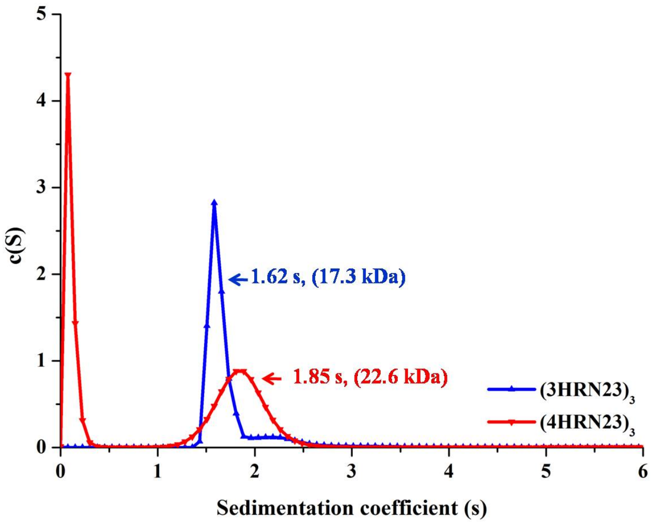

Fig. S6. Sedimentation velocity analysis of chimeric N-peptides.

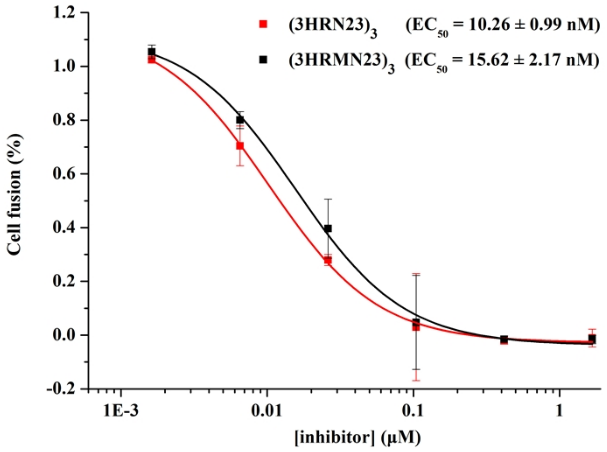

Fig. S7. Inhibition of HIV-1 Env-mediated cell-cell fusion using $(3HRN23)_3$ and $(3HRMN23)_3$. Data were derived from the results of three independent experiments. The numbers shown in parentheses are EC_{50} values.

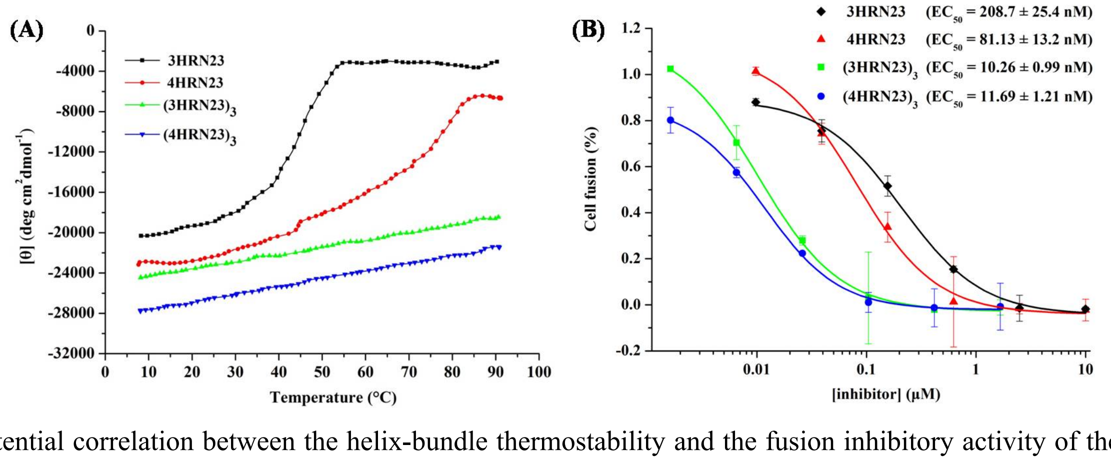

Fig. S8. Potential correlation between the helix-bundle thermostability and the fusion inhibitory activity of the chimeric N-peptides. (**A**) The thermostability of chimeric N-peptides. The final concentration of each peptide in PBS containing 4 M guanidinium chloride was 10 μM. (**B**) Inhibition of HIV-1 Env-mediated cell-cell fusion using chimeric N-peptides. Data were derived from the results of three independent experiments. The numbers shown in parentheses are EC_{50} values.

Materials and methods

Peptide synthesis. Peptides were synthesized using a Liberty™ automated microwave peptide synthesizer (CEM Co., Matthews, NC) with a standard solid-phase N-(9-fluorenyl)methoxycarbonyl (Fmoc) chemistry protocol. All protected amino acids used were purchased from GL Biochem Ltd. (Shanghai, China). Rink Amide resin (0.38–0.45 mmol/g, Nankai Hecheng S&T Co. Ltd., Tianjin, China) was used. Coupling of the amino acids was achieved using O-benzotriazol-1-yl-N,N,N',N'-tetramethyl-uronium hexafluorophosphate (HBTU, GL Biochem, Shanghai, China) and diisopropylethylamine (DIEA, Acrose) as an activator and an active base, respectively, in N,N-dimethylformamide (DMF) solution. The Fmoc protection group was removed using 20% piperidine/DMF. Between every coupling or Fmoc removal, the resin was washed five times with DMF and three times with dichloromethane (DCM). The carboxyl termini were amidated upon cleavage from the resin, and the amino termini were capped with acetic acid anhydride. For peptides possessing a side chain thioester, Fmoc-L-glutamic acid O-allyl ester [Fmoc-Glu(OAll)-OH] was used at the thioester-modified site. After all amino acids had coupled on the resin in the peptide synthesizer, the O-allyl group was removed manually by 1 eq tetrakis(triphenylphosphine)palladium with 10 eq 5,5-dimethyl-1,3-cyclohexanedione as scavenger in DCM/THF(1:1) solution. Then the resin was washed five times with 0.5% DIEA in DMF and five times with 1 M sodium diethyldithiocarbamate in DMF. 4 eq 1-Ethyl-3-(3-dimethyllaminopropyl)carbodiimide hydrochloride (EDC) and 4 eq benzyl mercaptan were added to the resin for thioester formation. The peptides were cleaved from the Rink Amide resin and deprotected with Reagent K, which contained 82.5% trifluoroacetic acid (TFA), 5% thioanisole, 5% m-cresol, 5% water, and 2.5% ethanedithiol. The crude peptide products were precipitated with cold diethyl ether, lyophilized, and purified by preparative reverse-phase high-performance liquid chromatography (HPLC) using a Waters preparative HPLC system (PrepLC 4000): gradient elution of 30–50% solvent B in solvent A (0.1% TFA in H_2O, solvent A; 0.1% TFA in 70% CH_3CN/H_2O, solvent B) over 60 min at 16 mL/min on a Waters X-bridge C8, 10 µm, 19.5 mm × 250 mm column. Analytical RP-HPLC was performed on a RP-C8 column (Zorbax Eclipse XDB-C8, 5 µm, 4.6 mm × 150 mm) with gradient elution of 5–100% solvent B in solvent A over 25 min at a flow rate of 1 mL/min. Compounds were detected by UV absorption at 210 nm with a Shimadzu SPD-10A detector. All peptides were purified to >95% purity. The molecular weight of the peptides was confirmed by matrix-assisted laser desorption/ionization-time of flight mass spectrometry (MALDI-TOF-MS; Autoflex III, Bruker Daltonics).

Isopeptide bond formation (interchain acyl transfer reaction). In brief, the purified thioester-peptide precursor (1.0 equiv., 0.005mmol) was dissolved in 1 mL of PBS/H_2O/CH_3CN (3:2:5 v/v) and stirred at room temperature. The reaction was monitored by analytical RP-HPLC and confirmed by MALDI-TOF-MS.

Tricine-sodium dodecyl sulfate-polyacrylamide gel electrophoresis (Tricine-SDS-PAGE). Polyacrylamide gels (12%) and a BayGene Mini Cell were used for Tricine-SDS-PAGE. The cathode buffer consisted of 0.1 M Tricine, 0.1 M Tris, and 1% SDS, and the anode buffer was 0.2 M Tris. After incubation at 37 °C for 0–40 h, the samples were mixed with Tris-SDS-glycine sample buffer (Invitrogen, Carlsbad, CA) at a ratio of 1:1 and then loaded onto the gels (20 μL/well). Gel electrophoresis was first carried out at a constant voltage of 60 V at room temperature for 1 h and then carried out at a constant voltage of 120 V at room temperature for 2 h. The gel was then stained with Bio-Rad Bio-Safe Coomassie Stain.

Proteolytic stability

Qualification assay. Chromatographic analyses were performed using an ODS-C8 column (5 μm, 100 mm × 2.0mm ID) kept at ambient temperature. The mobile phase was composed of acetonitrile-water-formic acid in the ratio of 60:40:0.1 (v/v/v) at a flow rate of 0.3 mL/min. The sample injection volume was 10 μL. Acetonitrile was HPLC grade, and other chemical reagents and solvents were analytical grade. A Thermo TSQ Quantum Discovery MAX triple–quadruple tandem mass spectrometer equipped with ESI source (San Jose, CA) and Surveyor LC pump were used for LC-MS/MS analysis. Data acquisition and data processing were performed by using Xcalibur software and LCQuan 2.0 data analysis program (Thermo Finnigan), respectively. Optimized MS parameters were as below: 4800 V spray voltage, 10.0 psi sheath gas pressure, 1.0 psi auxiliary valve flow, and 300 °C of capillary temperature. When running collision-induced dissociation (CID), the pressure was set to 1.5 mTorr. The selected ion monitoring (SIM) mode was preformed for T20 (*m*/*z* 1498 $[M+3H]^{3+}$) and $(3HRN23)_3$ (*m*/*z* 1327 $[M+13H]^{13+}$).

Preparation of Samples. By use of a simple protein precipitation method, a test compound was extracted from liver homogenate and 20 ng/mL proteinase K in PBS, respectively. Samples for standard curves were prepared by spiking 50 μL of rat liver homogenate or 20 ng/mL proteinase K with 100 μL of various concentrations of each test compound ranging from 0.1－5 μg/mL in ethanol. To each tested sample (50 μL), 200 μL ethanol was added. After the mixture was vortexed and centrifuged at 18000*g* for 10 min, the supernatant was transferred to auto-sampler vials, and 10 μL of the supernatant was injected into the LC-MS/MS system for analysis.

Assessment of in Vitro degradation. Three male Sprague-Dawley rats (250 ± 20 g) were anesthesia, sacrificed by collecting blood via abdominal aorta. The liver was quickly removed from each rat, washed in ice-cold 0.9% sodium chloride solution, weighed and cut into small pieces, diluted with 4 mL of ice-cold Tris–HCl buffer (50 mM, pH 7.4) per 1 g of tissue. For each tissue, the samples from the three rats were pooled and homogenized using a Teflon digital homogenizer. The homogenates were centrifuged (9,000g) for 20 min at 4°C. Test compounds were added to the homogenates or 20 ng/mL proteinase K in PBS at a final concentration of 10 μg/mL, and then the reaction was initiated

by heating at 37°C in a water bath. The samples (100 μL) were withdrawn at different time intervals (0, 30, 60 and 120min) and tested as described above.

MALDI-TOF-MS of designed peptides

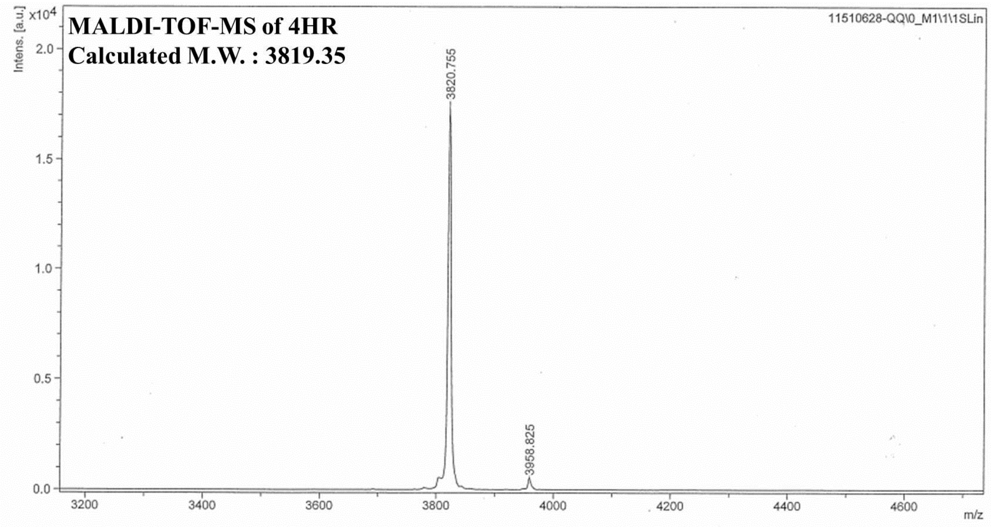

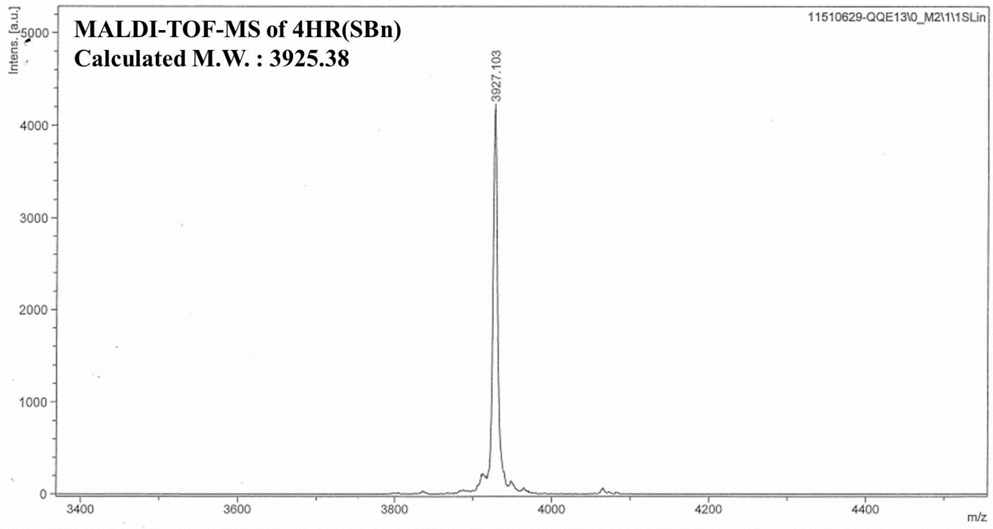

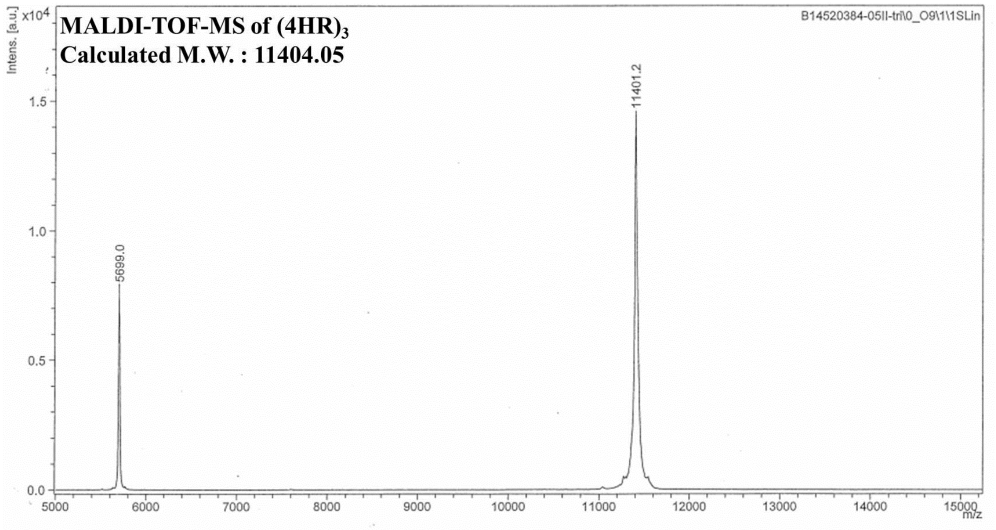

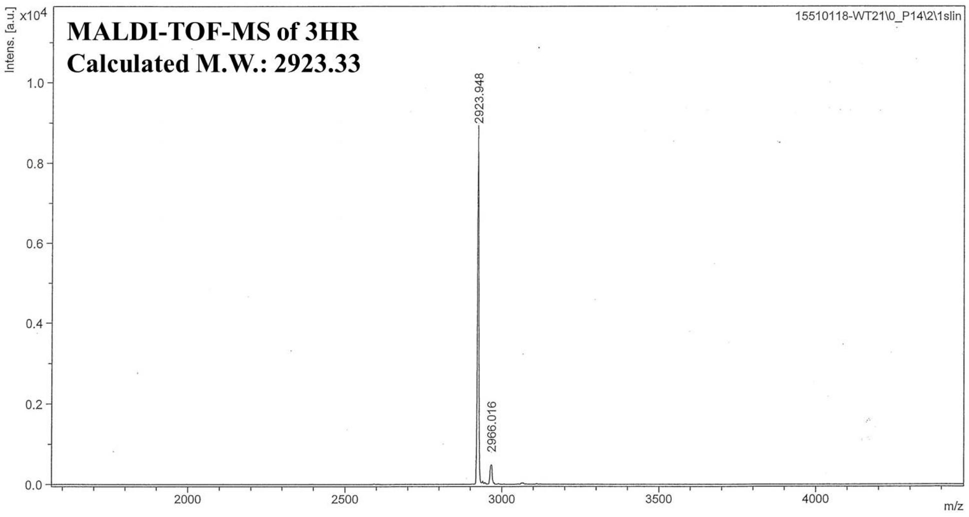
MALDI-TOF-MS of 3HR
Calculated M.W.: 2923.33
15510118-WT21\0_P14\2\1slin
Intens. [a.u.]
x10^4
2923.948
2966.016
m/z
1.0
0.8
0.6
0.4
0.2
0.0
2000
2500
3000
3500
4000

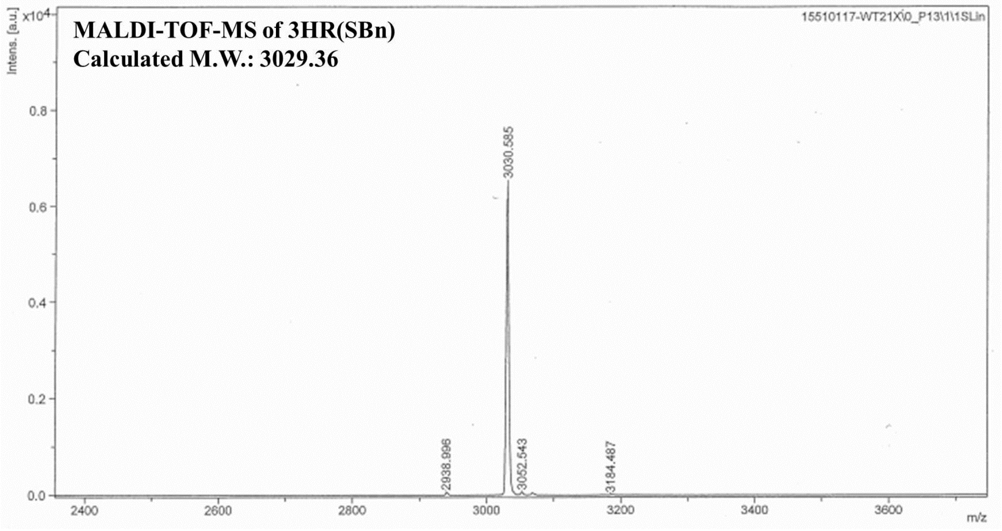
MALDI-TOF-MS of 3HR(SBn)
Calculated M.W.: 3029.36
15510117-WT21X\0_P13\1\1SLin
Intens. [a.u.]
x10^4
3030.585
2938.996
3052.543
3184.487
m/z
0.8
0.6
0.4
0.2
0.0
2400
2600
2800
3000
3200
3400
3600

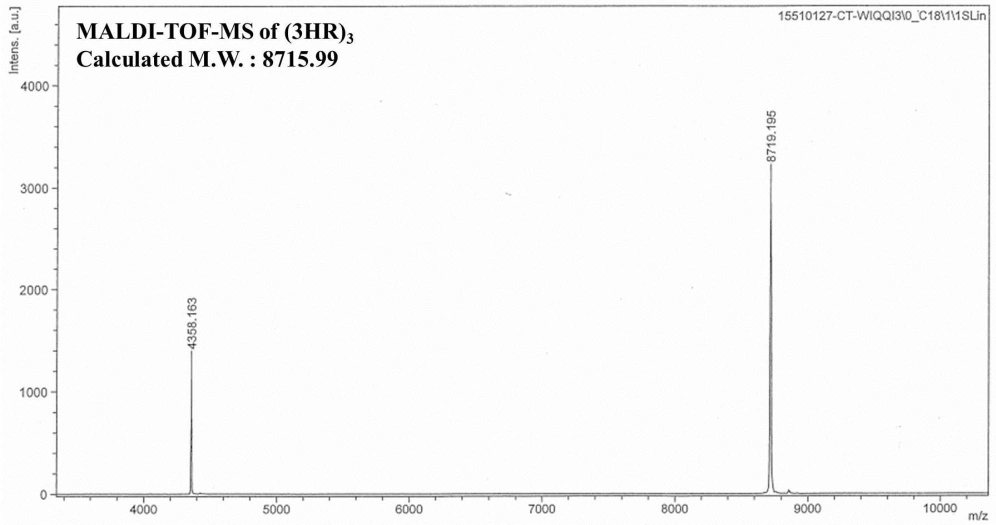
MALDI-TOF-MS of $(3HR)_3$
Calculated M.W. : 8715.99
15510127-CT-WIQQI3\0_C18\1\1SLin
Intens. [a.u.]
8719.195
4358.163
m/z
4000
3000
2000
1000
0
4000
5000
6000
7000
8000
9000
10000

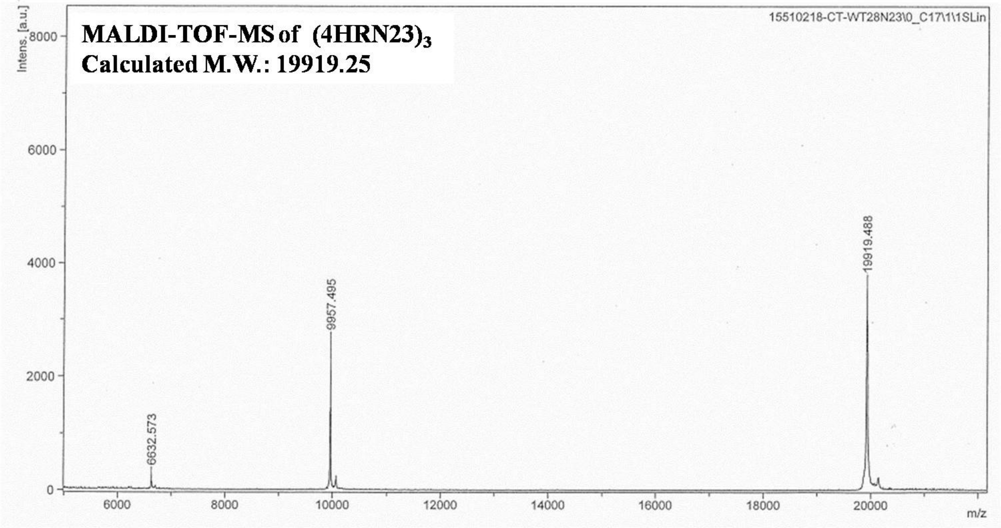
MALDI-TOF-MS of (4HRN23)3
Calculated M.W.: 19919.25
15510218-CT-WT28N23\0_C17\1\1SLin
Intens. [a.u.]
8000
6000
4000
2000
0
6632.573
9957.495
19919.488
6000
8000
10000
12000
14000
16000
18000
20000
m/z

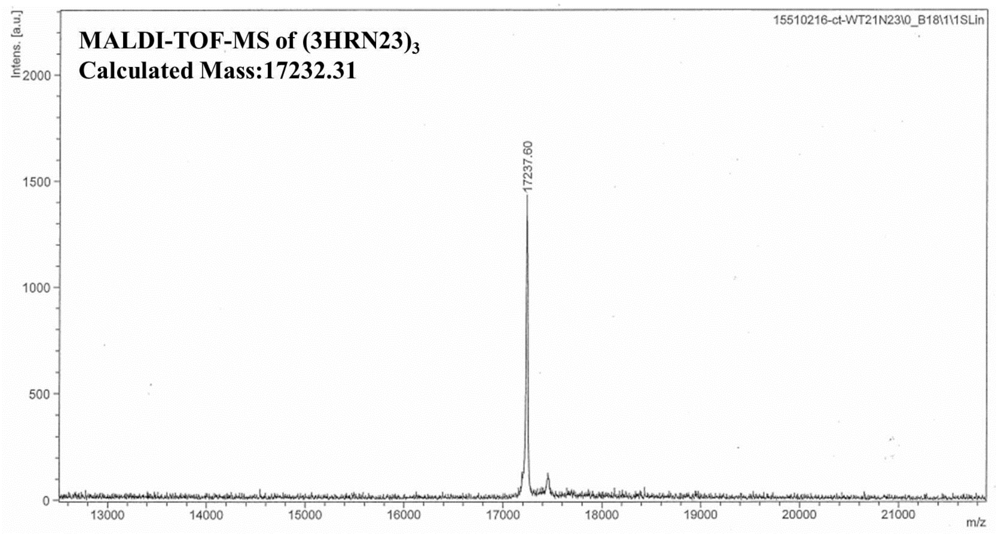
MALDI-TOF-MS of (3HRN23)3
Calculated Mass:17232.31
15510216-ct-WT21N23\0_B18\1\1SLin
Intens. [a.u.]
2000
1500
1000
500
0
17237.60
13000
14000
15000
16000
17000
18000
19000
20000
21000
m/z
